# Level 3 Cardiopulmonary Exercise Testing to Guide Therapeutic Decisions in Non-Severe Pulmonary Hypertension with Lung Disease

**DOI:** 10.3390/life15071089

**Published:** 2025-07-11

**Authors:** Raj Parikh, Chebly Dagher, Harrison W. Farber

**Affiliations:** 1 Division of Pulmonary, Critical Care and Sleep, Hartford Hospital, Hartford, CT 06102, USA; 2 Department of Internal Medicine, University of Connecticut, Farmington, CT 06030, USA; 3 Division of Pulmonary, Sleep and Critical Care Medicine, Tufts Medical Center, Boston, MA 02111, USA

**Keywords:** level 3 cardiopulmonary exercise testing, group 3 pulmonary hypertension, pulmonary hypertension, interstitial lung disease, pulmonary vascular resistance, treprostinil

## Abstract

Inhaled treprostinil is approved for the treatment of pulmonary hypertension-associated interstitial lung disease (PH-ILD); however, it has not shown significant benefit in patients with a pulmonary vascular resistance (PVR) < 4 WU. As such, treatment for non-severe PH-ILD remains controversial. A total of 16 patients with non-severe PH-ILD were divided into two groups based on changes in PVR during exercise: a dynamic PVR group (*n* = 10), characterized by an increase in PVR with exertion, and a static PVR group (*n* = 6), with no increase in PVR with exercise. The dynamic PVR group received inhaled treprostinil, while the static PVR group was monitored off therapy. Baseline and 16-week follow-up values were compared within each group. At 16 weeks, the dynamic PVR group demonstrated significant improvements in mean 6 min walk distance (6MWD) (+32.5 m, *p* < 0.05), resting PVR (−1.04 WU, *p* < 0.05), resting mean pulmonary arterial pressure (mPAP) (−5.8 mmHg, *p* < 0.05), exercise PVR (−1.7 WU, *p* < 0.05), exercise mPAP (−13 mmHg, *p* < 0.05), and estimated right ventricular systolic pressure (−9.2 mmHg, *p* < 0.05). In contrast, the static PVR group remained clinically stable. These observations suggest that an exercise-induced increase in PVR, identified through Level 3 CPET, may help select patients with non-severe PH-ILD who are more likely to benefit from early initiation of inhaled treprostinil.

## 1. Introduction

**Figure 1:**
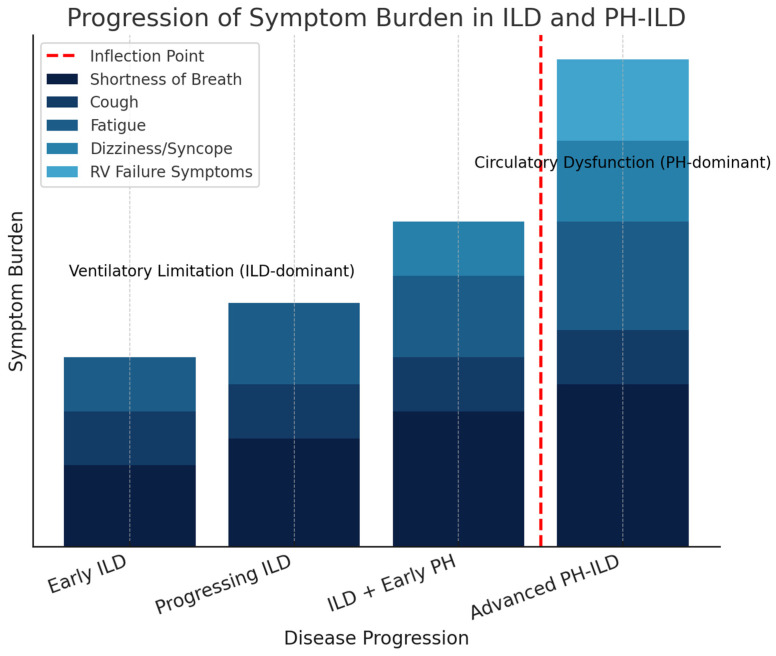
Transition from Ventilatory Limitation to Circulatory Dysfunction in PH-ILD.

Pulmonary hypertension (PH) associated with interstitial lung disease (PH-ILD) is a type of pre-capillary PH, defined by a mean pulmonary artery pressure (mPAP) of 20 mmHg or higher, a pulmonary capillary wedge pressure (PCWP) of 15 mmHg or lower, and a pulmonary vascular resistance (PVR) exceeding 2 Wood units (WUs) [1]. In PH-ILD, the underlying parenchymal lung disease and pulmonary vascular remodeling collectively drive disease progression, initially presenting as a ventilatory limitation but eventually leading to hemodynamic impairment and right ventricular (RV) dysfunction [1,2]. Patients with PH-ILD initially experience breathlessness primarily due to ventilatory limitations from the underlying ILD [2]. However, as the disease progresses, they reach a point at which circulatory dysfunction, driven by PH and subsequent RV impairment, becomes the dominant factor in their symptoms, significantly impacting quality of life [3,4]. Identifying patients before this transition point is crucial, as timely treatment initiation may help delay it, preventing PH and RV dysfunction from becoming the primary drivers of disease progression (Figure 1).

Figure 1 illustrates the progression of symptom burden in patients with PH-ILD over time. In the early stages, symptoms are primarily driven by ventilatory limitations due to the underlying interstitial lung disease (ILD). As the disease advances, pulmonary hypertension (PH) and right ventricular (RV) dysfunction contribute to worsening symptoms, leading to circulatory dysfunction. The red dashed line marks the inflection point, where circulatory impairment surpasses ventilatory limitation as the primary driver of symptoms.

PH-ILD can be classified as severe (PVR > 5 WU) or non-severe (PVR ≤ 5 WU), with a PVR > 5 WU being a strong predictor of poor prognosis [1,5]. Inhaled treprostinil, a prostacyclin analog, received FDA approval for PH-ILD based on the INCREASE trial that demonstrated significant clinical benefits [6,7]. However, a pre-specified analysis within the INCREASE trial showed that patients with a baseline PVR < 4 WU did not achieve a significant improvement in six-minute walk distance (6MWD) with inhaled treprostinil, suggesting that baseline hemodynamics may influence treatment efficacy [6,8]. In contrast, post hoc analysis of the open-label extension of the INCREASE trial suggested that early treatment initiation in this population may reduce hospitalization risk and lower the likelihood of lung disease exacerbation, although these findings were not statistically significant [9]. Therefore, more detailed data on which patients should undergo early treatment is lacking and important.

Level 3 cardiopulmonary exercise testing (CPET) provides a detailed assessment of pulmonary vascular function during exercise [10,11]. Unlike resting hemodynamics, a Level 3 CPET can measure exercise-induced changes in PVR, offering insights into disease severity and functional impairment [11,12]. In this study, we used the PVR response during Level 3 CPET in 16 patients with non-severe PH-ILD (PVR < 5 WU) [1] to guide treatment decisions.

## 2. Materials and Methods

This retrospective, observational cohort study included 16 World Health Organization (WHO) Group 3 PH-ILD patients with a PVR of < 5 WU, none of whom were receiving PH therapy. The diagnosis of PH was confirmed by right heart catheterization (RHC); diagnosis of ILD was identified by diffuse parenchymal lung disease on computed tomography (CT). All patients underwent Level 3 CPET using a sitting cycle ergometer under constant-load conditions, with continuous monitoring. Based on hemodynamic response, patients were stratified into two cohorts: a static PVR group (PVR_S_, *n* = 6), in whom there was no change in PVR during exercise (defined as no increase in exercise PVR compared to resting values) and treatment was deferred, and a dynamic PVR group (PVR_D_, *n* = 10), in whom there was an increase in PVR during exercise and treatment was initiated with inhaled treprostinil. Treprostinil was titrated based on individual tolerance, with an increase of 1 breath 4 times daily permitted every 3 days. The target dose was 9 breaths 4 times daily, with a maximum of 12 breaths 4 times daily. Dose adjustments were made by investigators to achieve the highest tolerated dose, in accordance with our standard clinical practice.

Baseline characteristics, including age, gender, ILD subtype, use of supplemental oxygen, and anti-fibrotic therapy, were recorded. Pulmonary function tests (PFTs), including predicted forced vital capacity (FVC) and diffusing capacity for carbon monoxide (DLCO), were also collected. Key hemodynamic and clinical parameters, including resting and exercise mPAP and PVR, 6MWD, echocardiogram-estimated right ventricular systolic pressure (eRVSP), and WHO Functional Class (WHO-FC), were assessed at baseline and after 16 weeks in both groups. The 16-week follow-up period was chosen based on the INCREASE study [6]. Descriptive statistics were used to summarize baseline characteristics. Within-group comparisons between baseline and 16-week follow-up values were performed using paired two-tailed *t*-tests. Statistical significance was defined as a *p*-value < 0.05. All analyses were conducted using Python 3.11. This study used convenience sampling. A formal sample size calculation was not performed, as this was a retrospective, exploratory study based on all eligible patients seen during the study period. The sample size reflects the full cohort of patients who met inclusion criteria and completed both baseline and 16-week follow-up assessments. The study was approved by the Hartford Hospital Institutional Review Board.

## 3. Results

The PVR_S_ group consisted of six patients with a mean age of 68.5 years (57–75), and was predominantly male. The most common subtype of ILD was usual interstitial pneumonia (UIP), with one case each of nonspecific interstitial pneumonia (NSIP) and combined pulmonary fibrosis and emphysema (CPFE). All patients were using supplemental oxygen, and three were receiving anti-fibrotic therapy. Baseline characteristics showed a mean resting PVR of 3.1 WU (3.0–3.3) and a mean resting mPAP of 23 mmHg (21–24). During exercise, the mean peak PVR was 2.8 WU (2.6–3.1) and mean peak mPAP was 38.2 mmHg (32–47). These patients did not have a significant change in exercise PVR. In this cohort, treatment was deferred. Additional baseline parameters included an average 6MWD of 356 m (270–400), eRVSP of 42 mmHg (38–50), FVC of 67% predicted (60–70%), and DLCO of 43.5% predicted (33–52%) (Table 1).

Table 1 presents the baseline hemodynamic and clinical measurements of patients with static pulmonary vascular resistance (PVR) after level 3 cardiopulmonary exercise testing (CPET). Age is reported in years, and sex is categorized as male or female. The type of interstitial lung disease (ILD) is specified for each patient, with common subtypes including usual interstitial pneumonia (UIP), nonspecific interstitial pneumonia (NSIP), and combined pulmonary fibrosis and emphysema (CPFE). The table also indicates whether patients required supplemental oxygen or were receiving antifibrotic therapy at baseline. Hemodynamic parameters include resting mean pulmonary arterial pressure (mPAP) in millimeters of mercury (mmHg) and resting pulmonary vascular resistance (PVR) in Wood units (WUs), along with peak values for mPAP and PVR during exercise testing. Functional capacity is assessed through the six-minute walk distance (6MWD), measured in meters, and estimated right ventricular systolic pressure (eRVSP) in mmHg. Additionally, the World Health Organization (WHO) Functional Class (FC) is reported. Pulmonary function is represented by forced vital capacity (FVC) and diffusing capacity for carbon monoxide (DLCO), both expressed as a percentage of the predicted values.

**Figure 2:**
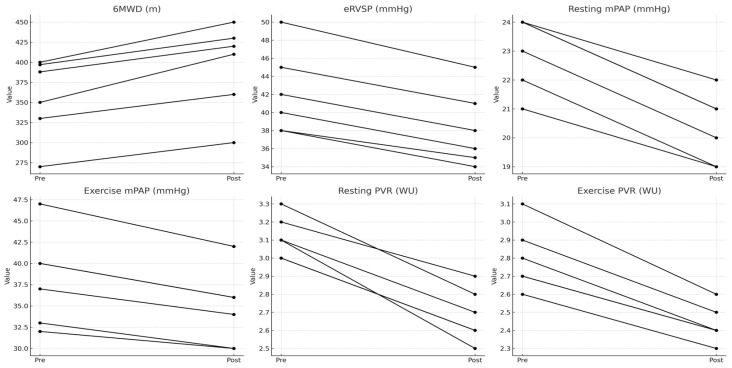
Changes in Functional and Hemodynamic Parameters in PVR_S_ Group Over 16 Weeks.

After 16 weeks, the PVR_S_ group, despite not having been treated, maintained stable hemodynamic and functional profiles, with no statistically significant changes in resting mPAP (mean 24 mmHg, *p* = 0.15), resting PVR (mean 3.1 WU, *p* = 0.61), exercise mPAP (mean 39 mmHg, *p* = 0.15, and exercise PVR (mean 2.9 WU, *p* = 0.25). The 6MWD remained comparable at 351 m (*p* = 0.28), while eRVSP was 42.5 mmHg (*p* = 0.69). All patients maintained WHO FC except for one patient who experienced worsening from FC I to FC II (Table 2 and Table 3; Figure 2).

Table 2 presents hemodynamic and clinical measurements of patients with static pulmonary vascular resistance (PVR) after level 3 cardiopulmonary exercise testing (CPET) after 16 weeks without treatment. Hemodynamic parameters include resting mean pulmonary arterial pressure (mPAP) in millimeters of mercury (mmHg) and resting PVR in Wood units (WUs), along with peak values for mPAP and PVR during exercise testing. Functional capacity is assessed through the six-minute walk distance (6MWD), measured in meters, and estimated right ventricular systolic pressure (eRVSP) in mmHg. The World Health Organization (WHO) Functional Class (FC) is also reported, indicating any changes in disease severity over the 16-week period.

Table 3 presents the mean baseline and 16-week follow-up values for hemodynamic and clinical parameters in the Static PVR group, which included patients with stable pulmonary vascular resistance (PVR) during level 3 cardiopulmonary exercise testing (CPET). These patients did not receive treatment and were monitored over time. The table includes resting and exercise mean pulmonary arterial pressure (mPAP) in mmHg, resting and exercise pulmonary vascular resistance (PVR) in Wood units (WUs), and estimated right ventricular systolic pressure (eRVSP) in mmHg. Functional capacity is assessed through the six-minute walk distance (6MWD) in meters. Values in parentheses represent the range (minimum–maximum), and *p*-values indicate statistical significance, with a threshold of *p* < 0.05 considered significant.

**Figure 3:**
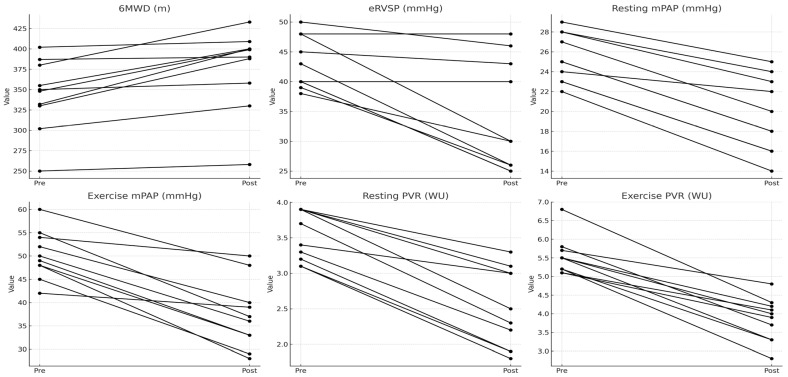
Changes in Functional and Hemodynamic Parameters in PVR_D_ Group Over 16 Weeks.

The PVR_D_ group consisted of ten patients with an average age of 67 years (55–77), and was predominantly male. Similar to the PVR_S_ group, the most common subtype of ILD was UIP, with one case of hypersensitivity pneumonitis (HP), one case of post-Coronavirus Disease ILD, and two cases of CPFE. All patients were using supplemental oxygen, and three were receiving anti-fibrotic therapy. Baseline hemodynamic assessments demonstrated a mean resting mPAP of 25.4 mmHg (22–29) and a mean resting PVR of 3.54 WU (3.1–3.9); during the CPET, there was a mean peak mPAP of 50.3 mmHg (42–60) and a mean peak PVR of 5.54 WU (5.1–6.8). In these patients, inhaled treprostinil was initiated. Additional baseline parameters included an average 6MWD of 344 m (250–402), eRVSP of 43.1 mmHg (38–50), mean FVC of 65% predicted (58–73%), and mean DLCO of 43% predicted (24–55%) (Table 4). After 16 weeks of treatment with inhaled treprostinil (average dose: 9 breaths four times a day; range: 9–12 breaths), the PVR_D_ group demonstrated significant improvements in hemodynamic and functional parameters. Statistical analysis revealed that mean resting mPAP decreased from 25.4 mmHg (22–29) to 19.6 mmHg (14–25) (*p* < 0.05) and mean resting PVR decreased from 3.54 WU (3.1–3.9) to 2.5 WU (1.8–3.3) (*p* < 0.05). Similarly, exercise testing showed a decrease in mean peak mPAP from 50.3 mmHg (42–60) to 37.3 mmHg (28–50) (*p* < 0.05) and a decrease in mean peak PVR from 5.54 WU (5.1–6.8) to 3.84 WU (2.8–4.8) (*p* < 0.05). Functionally, the mean 6MWD increased from 344 m (250–402) to 377 m (258–433) (*p* < 0.05), and the mean eRVSP decreased from 43.1 mmHg (38–50) to 33.9 mmHg (25–48) (*p* < 0.05). Additionally, the WHO FC distribution shifted significantly toward improvement, with more patients achieving FC I or asymptomatic status (Table 4 and Table 5; Figure 3).

Table 4 presents hemodynamic and clinical measurements of patients with dynamic pulmonary vascular resistance (PVR) after level 3 cardiopulmonary exercise testing (CPET) after 16 weeks of treatment with inhaled treprostinil. The inhaled treprostinil dose is reported as the number of breaths administered per session, four times daily. Hemodynamic parameters include resting mean pulmonary arterial pressure (mPAP) in millimeters of mercury (mmHg) and resting pulmonary vascular resistance (PVR) in Wood units (WUs), along with peak values for mPAP and PVR during exercise testing. Functional capacity is assessed through the six-minute walk distance (6MWD), measured in meters, and estimated right ventricular systolic pressure (eRVSP) in mmHg. The World Health Organization (WHO) Functional Class (FC) is also reported.

Table 5 presents the hemodynamic and functional parameter changes in the dynamic group at baseline and after 16 weeks of inhaled treprostinil therapy. The data include mean values for resting and exercise pulmonary vascular resistance (PVR) and mean pulmonary arterial pressure (mPAP), six-minute walk distance (6MWD), estimated right ventricular systolic pressure (eRVSP), and WHO Functional Class. Mean differences and *p*-values from paired *t*-tests are provided to assess statistical significance, with *p*-values < 0.01 indicating highly significant improvements.

Interestingly, both cohorts had comparable baseline characteristics, with no statistically significant overall differences except for baseline PVR and mPAP, which, although statistically different, do not seem clinically meaningful. The mean baseline PVR was 3.1 mmHg in the PVR_D_ group and 3.5 mmHg in the PVR_S_ group, while the mean mPAP was 23 mmHg and 25 mmHg, respectively (Table 6).

This table presents the baseline characteristics of patients categorized into Static Exercise PVR (*n* = 6) and Dynamic Exercise PVR (*n* = 10) groups. Variables include age, gender distribution, use of oxygen and antifibrotic therapy, and six-minute walk distance (6MWD). Data are expressed as mean ± standard deviation or as counts with percentages. *p*-values indicate statistical comparisons between the two groups, with no significant differences observed (*p* > 0.05).

## 4. Discussion

PH-ILD is classified as non-severe or severe based on hemodynamic parameters, with a PVR > 5 WU being a strong predictor of poor prognosis in PH-ILD [1,13]. Current guidelines define non-severe PH as PVR ≤ 5 WU and severe PH as PVR > 5 WU [13,14]. The INCREASE trial was the first to demonstrate that inhaled treprostinil improves exercise capacity in PH-ILD, with secondary analyses suggesting additional benefits such as improved FVC in specific subtypes of ILD and fewer disease progression events [6]. In patients with a baseline PVR < 4 WU, no significant difference in the primary endpoint (6MWD) was observed between those treated with inhaled treprostinil and those receiving placebo [6]. More broadly, treatment decisions for patients with non-severe PH-ILD and mild hemodynamic impairment remain poorly defined. It is still debated whether early initiation of inhaled treprostinil offers benefit or whether treatment should be reserved for more advanced disease. Observational data suggesting reductions in exacerbations, disease progression, cardiac biomarkers (e.g., NT-proBNP), or cardiopulmonary hospitalizations with early treatment are limited by small sample sizes and lack statistical validation and should therefore be interpreted with caution [8,9]. These uncertainties highlight the need for further research to clarify the role of early treatment in patients with milder disease. In an effort to explore this question, we examined whether Level 3 CPET could help identify treatment candidates among patients with non-severe PH-ILD by stratifying them into two groups, those with dynamic versus static pulmonary vascular responses to exercise, essentially distinguishing patients with more severe hemodynamic impairment during exertion. Patients in the dynamic group were treated with inhaled treprostinil, while those in the static group were monitored over the same period without intervention.

Both groups had very similar baseline characteristics. At 16-week follow-up, the PVR_D_ group demonstrated statistically significant improvements in multiple parameters, including PVR, mPAP, 6MWD, eRVSP, and WHO FC. Notably, 6MWD, the primary outcome of the INCREASE trial, improved in all 10 patients, with a mean increase of 32.5 m (from 344 m (250–402) to 376.5 m (258–433), *p* < 0.05). Hemodynamically, both resting and exercise mPAP and PVR significantly decreased, with a corresponding reduction in eRVSP. These findings further support the potential benefit of inhaled treprostinil in patients with more advanced disease, in this case reflected by a significant increase in PVR during Level 3 CPET. In contrast, the PVR_S_ group, in whom treatment was deferred, remained clinically stable with no significant changes at follow-up.

Early treatment in the PVR_D_ group appeared to result in favorable outcomes. This approach may aid in optimizing treatment selection and potentially delay disease progression to the point where circulatory dysfunction supersedes ventilatory limitation. Notably, worsening PVR during exercise, a potential marker of more advanced pulmonary vascular disease, may justify early intervention, even in the absence of severe resting hemodynamics. These findings underscore the need for future studies comparing therapeutic outcomes with inhaled treprostinil in both PVR_S_ and PVR_D_ groups, with both cohorts receiving treatment. Such studies could help confirm whether early initiation of therapy is beneficial in patients with mild resting hemodynamic impairment who exhibit worsening pulmonary vascular response during exercise. Additionally, they may clarify whether patients with static exercise PVR should remain under observation or might also benefit from treatment, despite previous large trials not demonstrating a significant effect in this population. In addition, another important question is whether the response to treatment correlates with the degree of worsening in exercise-induced hemodynamics. Moreover, several additional limitations must be acknowledged. The small sample size and single-center design limit the generalizability of our findings. The 16-week follow-up period may be insufficient to assess long-term outcomes, particularly in the PVR_S_ group. The absence of randomization and a control arm makes it challenging to attribute observed improvements solely to inhaled treprostinil. Additionally, Level 3 CPET requires maximal effort and may not be feasible or well-tolerated in all patients, which may limit its applicability in certain clinical settings. Recent work by Wiecha et al. showed that submaximal CPET can reliably estimate VO_2_max in healthy individuals, offering a less demanding alternative to maximal testing. While our study required maximal effort, submaximal approaches may warrant further exploration in patients with limited exercise tolerance [15]. Furthermore, while the use of exercise-induced PVR for risk stratification is promising, it is not yet formally validated. Lastly, the study focused on hemodynamic and functional changes, without evaluating key long-term clinical outcomes such as survival or hospitalization rates.

## 5. Conclusions

In our cohort, patients with a dynamic PVR response during exercise, as assessed by Level 3 CPET, demonstrated significant functional and hemodynamic improvement after treatment with inhaled treprostinil, while those with a static PVR response remained clinically stable off therapy. These findings suggest that Level 3 CPET may provide important physiologic insights beyond resting hemodynamics and help guide more individualized treatment strategies in non-severe PH-ILD. The management of PH-ILD remains challenging, and the optimal approach for treating patients with mild hemodynamic impairment is still controversial. Although treatment decisions should be based on a combination of clinical, physiological, and hemodynamic factors, further prospective studies are needed to validate this approach and assess long-term outcomes.

## Figures and Tables

**Table 1:** Baseline Hemodynamic and Clinical Measurements of Patients with static PVR after Level 3 CPET.

Patient	Age	Sex	ILD	On O2	On Antifibrotic Therapy	Baseline Resting mPAP	Baseline Resting PVR	Baseline Exercise mPAP (Peak)	Baseline Exercise PVR (Peak)	Baseline 6MWD	Baseline eRVSP on TTE	Baseline WHO FC	Baseline FVC	Baseline DLCO
1	57	F	UIP	Y	Y	24	3.1	33	2.8	350	42	2	66	44
2	71	F	UIP	Y	Y	24	3.3	40	3.1	330	45	2	60	40
3	67	M	NSIP	Y	N	21	3	32	2.7	400	38	1	70	50
4	75	M	CPFE	Y	N	24	3.2	47	2.9	270	50	3	65	33
5	70	M	UIP	Y	Y	23	3.1	37	2.6	388	40	1	69	52
6	71	M	UIP	Y	N	22	3	40	2.7	397	38	1	70	42

**Table 2:** Hemodynamic and Clinical Measurements After 16 Weeks of Follow-Up in Patients with Static PVR on Level 3 CPET Who Were Monitored Off Treatment.

Patient	Resting mPAP	Resting PVR	Exercise mPAP (Peak)	Exercise PVR (Peak)	6MWD	eRVSP	WHO FC
1	25	3.1	34	2.8	355	44	2
2	24	3.3	44	2.9	332	45	2
3	20	2.9	33	2.7	397	40	1
4	28	3.3	49	3.3	245	48	3
5	24	3.1	37	2.9	385	42	1
6	24	3.1	39	2.9	390	36	2

**Table 3:** Hemodynamic and Functional Parameter Changes in the Static Group: Baseline vs. 16-Week Follow-Up.

Variable	Baseline Mean	16-Week Follow-Up Mean	Mean Difference	*p* Value
Resting mPAP	23	24.17	1.17	0.15
Resting PVR	3.12	3.13	0.02	0.61
Exercise mPAP (peak)	38.17	39.33	1.17	0.15
Exercise PVR (peak)	2.8	2.92	0.12	0.25
6MWD	356	351	-5	0.28
eRSVP	42.1	42.5	0.4	0.69

**Table 4:** Hemodynamic and Clinical Measurements After 16 Weeks of Follow-Up in the Dynamic PVR Group Treated with Inhaled Treprostinil.

Patient	Age	Sex	ILD	On O2	On Antifibrotic Therapy	Baseline Resting mPAP	Baseline Resting PVR	Baseline Exercise mPAP (Peak)	Baseline Exercise PVR (Peak)	Baseline 6MWD	Baseline eRVSP	Baseline WHO FC	Baseline FVC (%)	Baseline DLCO (%)
1	55	F	UIP	Yes	Yes	25	3.7	45	5.1	355	43	1	72	42
2	67	M	UIP	Yes	Yes	27	3.9	49	5.5	330	48	2	68	49
3	62	M	UIP	Yes	No	23	3.1	48	5.2	402	40	2	62	48
4	59	M	UIP	Yes	Yes	28	3.9	52	5.5	350	45	3	73	55
5	66	F	HP	Yes	No	24	3.4	42	5.1	387	40	2	70	53
6	73	M	Post-COVID	Yes	No	25	3.3	55	5.8	380	38	1	63	44
7	74	M	CPFE	Yes	No	28	3.9	60	6.8	302	50	2	59	24
8	69	M	CPFE	Yes	No	29	3.9	54	5.7	250	48	3	58	33
9	71	F	UIP	Yes	No	22	3.1	50	5.5	332	39	1	62	40
10	77	M	UIP	Yes	No	23	3.2	48	5.2	348	40	1	64	41

**Table 5:** Hemodynamic and Functional Parameter Changes in the Dynamic Group: Baseline vs. 16-Week Follow-Up.

Variable	Baseline Mean	16-Week Follow-Up Mean	Mean Difference	*p* Value
Resting PVR	3.54	2.5	−1.04	<0.01
Exercise mPAP (peak)	50.3	37.3	−13	<0.01
Exercise PVR (peak)	5.54	3.84	−1.7	<0.01
6MWD	344	377	33	<0.01
eRVSP	43.1	33.9	−9.2	<0.01

**Table 6:** Differences in Baseline Characteristics Between Patients with Static and Dynamic Exercise PVR.

Baseline Characteristics	Static Exercice PVR (N = 6)	Dynamic Exercice PVR (N = 10)	*p* Value
Age (years)	68.5 ± 6	67 ± 7	>0.05
Male gender, *n* (%)	4 (67%)	7 (70%)	>0.05
Oxygen therapy, *n* (%)	6 (100%)	10 (100%)	>0.05
Antifibrotic therapy, *n* (%)	2 (33%)	3 (30%)	>0.05
6MWD (m)	356 ± 50	344 ± 44	>0.05
FVC (% predicted)	68 ± 5	65 ± 5	>0.05
DLCO (% predicted)	44 ± 7	43 ± 9	>0.05
eRVSP (mmHg)	42 ± 5	43 ± 4	>0.05
Resting mPAP	23 ± 1	25 ± 2	<0.05
Resting PVR	3.1 ± 0.12	3.5 ± 0.35	<0.05

## Data Availability

The original contributions presented in this study are included in the article. Further inquiries can be directed to the corresponding author.

## Abbreviations

The following abbreviations are used in this manuscript:

PH	pulmonary hypertension
ILD	interstitial lung disease
PH-ILD	pulmonary hypertension associated interstitial lung disease
PVR	pulmonary vascular resistance
WU	Wood units
CPET	cardiopulmonary exercise testing
mPAP	mean pulmonary arterial pressure
eRVSP	estimated right ventricular systolic pressure
PCWP	pulmonary capillary wedge pressure
RV	right ventricular
6MWD	six-minute walk distance
RHC	right heart catheterization
WHO	World Health Organization
FC	functional class
WHO-FC	World Health Organization Functional Class
CT	computed tomography
PVRS	non-significant change in pulmonary vascular resistance during exercise
PVRD	increase in pulmonary vascular resistance during exercise
PFTs	Pulmonary function tests
FVC	forced vital capacity
DLCO	diffusing capacity for carbon monoxide
UIP	usual interstitial pneumonia
NSIP	nonspecific interstitial pneumonia
CPFE	combined pulmonary fibrosis and emphysema
PDE5	Phosphodiesterase-5

## References

[1] Humbert M., Kovacs G., Hoeper M.M., Badagliacca R., Berger R.M., Brida M., Carlsen J., Coats A.J., Escribano-Subias P., Ferrari P. (2022). 2022 ESC/ERS Guidelines for the diagnosis and treatment of pulmonary hypertension: Developed by the task force for the diagnosis and treatment of pulmonary hypertension of the European Society of Cardiology (ESC) and the European Respiratory Society (ERS). Endorsed by the International Society for Heart and Lung Transplantation (ISHLT) and the European Reference Network on rare respiratory diseases (ERN-LUNG). Eur. Heart J..

[2] Nikkho S.M., Richter M.J., Shen E., Abman S.H., Antoniou K., Chung J., Fernandes P., Hassoun P., Lazarus H.M., Olschewski H. (2022). Clinical significance of pulmonary hypertension in interstitial lung disease: A consensus statement from the Pulmonary Vascular Research Institute’s innovative drug development initiative—Group 3 pulmonary hypertension. Pulm. Circ..

[3] King C.S., Shlobin O.A. (2020). The trouble with group 3 pulmonary hypertension in interstitial lung disease: Dilemmas in diagnosis and the conundrum of treatment. Chest.

[4] Braganza M., Shaw J., Solverson K., Vis D., Janovcik J., Varughese R.A., Thakrar M.V., Hirani N., Helmersen D., Weatherald J. (2019). A prospective evaluation of the diagnostic accuracy of the physical examination for pulmonary hypertension. Chest.

[5] Krompa A., Marino P. (2023). Diagnosis and management of pulmonary hypertension related to chronic respiratory disease. Breathe.

[6] Waxman A., Restrepo-Jaramillo R., Thenappan T., Ravichandran A., Engel P., Bajwa A., Allen R., Feldman J., Argula R., Smith P. (2021). Inhaled treprostinil in pulmonary hypertension due to interstitial lung disease. N. Engl. J. Med..

[7] McEvoy C., Argula R., Sahay S., Shapiro S., Eagan C., Hickey A.J., Smutney C., Dillon C., Winkler T., Davis B.N. (2023). Tyvaso DPI: Drug-device characteristics and patient clinical considerations. Pulm. Pharmacol. Ther..

[8] Waxman A., Restrepo R., Thenappan T., Engel P., Bajwa A., Ravichandran A., Feldman J., Case A., Tapson V., Smith P. Long-Term effects of inhaled treprostinil in patients with pulmonary hypertension due to interstitial lung disease: The increase study open-label extension. Proceedings of the American Thoracic Society Conference.

[9] Weatherald J., Nathan S.D., El-Kersh K., Argula R.G., DuBrock H.M., Rischard F.P., Cassady S.J., Tarver J., Levine D.J., Tapson V.F. (2024). Inhaled treprostinil in patients with pulmonary hypertension associated with interstitial lung disease with less severe haemodynamics: A post hoc analysis of the INCREASE study. BMJ Open Respir. Res..

[10] Guazzi M., Adams V., Conraads V., Halle M., Mezzani A., Vanhees L., Arena R., Fletcher G.F., Writing Committee, EACPR, AHA (2012). Clinical recommendations for cardiopulmonary exercise testing data assessment in specific patient populations. Eur. Heart J..

[11] Farina S., Correale M., Bruno N., Paolillo S., Salvioni E., Badagliacca R., Agostoni P. (2018). The role of cardiopulmonary exercise tests in pulmonary arterial hypertension. Eur. Respir. Rev..

[12] Vaidy A., Vahdatpour C.A., Mazurek J. (2024). Exercise Testing in Patients with Pulmonary Hypertension. J. Clin. Med..

[13] Olsson K.M., Hoeper M.M., Pausch C., Grünig E., Huscher D., Pittrow D., Rosenkranz S., Gall H. (2021). Pulmonary vascular resistance predicts mortality in patients with pulmonary hypertension associated with interstitial lung disease: Results from the COMPERA registry. Eur. Respir. J..

[14] Zeder K., Avian A., Bachmaier G., Douschan P., Foris V., Sassmann T., Troester N., Brcic L., Fuchsjaeger M., Marsh L.M. (2021). Elevated pulmonary vascular resistance predicts mortality in COPD patients. Eur. Respir. J..

[15] Wiecha S., Kasiak P.S., Szwed P., Kowalski T., Cieśliński I., Postuła M., Klusiewicz A. (2023). VO2max prediction based on submaximal cardiorespiratory relationships and body composition in male runners and cyclists: A population study. Elife.

